# Spina Bifida Prevention: A Narrative Review of Folic Acid Supplements for Childbearing Age Women

**DOI:** 10.7759/cureus.53008

**Published:** 2024-01-26

**Authors:** Lloyd F Ledet III, Connor J Plaisance, Charles P Daniel, Maxwell J Wagner, Ivan Alvarez, Caroline R Burroughs, Ross Rieger, Harish Siddaiah, Shahab Ahmadzadeh, Sahar Shekoohi, Alan D Kaye, Giustino Varrassi

**Affiliations:** 1 School of Medicine, Louisiana State University Health Sciences Center, Shreveport, USA; 2 Department of Anesthesiology, Louisiana State University Health Sciences Center, Shreveport, USA; 3 Department of Pain Medicine, Paolo Procacci Foundation, Rome, ITA

**Keywords:** folate, folic acid, spina bifida, neural tube defects, neural tube development

## Abstract

Neural tube defects (NTDs) are malformations that occur during embryonic development, and they account for most central nervous system birth anomalies. Genetic and environmental factors have been shown to play a role in the etiology of NTDs. The different types of NTDs are classified according to anatomic location and severity of the defect, with most of the neural axis anomalies occurring in the caudal spinal or cranial areas. Spina bifida is a type of NTD that is characterized by an opening in the vertebral arch, and the level of severity is determined by the extent to which the neural tissue protrudes through the opened arch(es). Prevention of NTDs by administration of folic acid has been studied and described in the literature, yet there are approximately 300,000 cases of NTDs that occur annually, with 88,000 deaths occurring per year worldwide. A daily intake of at least 400 μg of folic acid is recommended especially for women of childbearing age. To provide the benefits of folic acid, prenatal vitamins are recommended in pregnancy, and many countries have been fortifying foods such as cereal grain products with folic acid; however, not all countries have instituted folic acid fortification programs. The present investigation includes a description of the pharmacology of folic acid, neural tube formation, defects such as spina bifida, and the relevance of folic acid to developing spina bifida. Women’s knowledge and awareness of folic acid regarding its importance in the prevention of spina bifida is a major factor in reducing incidence worldwide.

## Introduction and background

Neural tube defects (NTDs) are malformations that occur during embryonic development and account for most central nervous system birth anomalies. The different types of NTDs are classified based on the anatomic location and severity of the defect [1]. Most of the neural axis anomalies occur in the caudal spinal or cranial areas [2,3]. With an incidence of 0.1% in the Western world and 1.86% worldwide, there are approximately 300,000 cases of NTDs that occur annually [4-6], with 88,000 deaths occurring per year worldwide [7].

Spina bifida is a type of NTD characterized by an opening in the vertebral arch through which there can be a herniation of meninges, cystic masses, spinal cord, and nerves. There are varying levels of severity, which are determined by the extent to which the neural tissue protrudes through the opened arch(es) [8]. Potential disabilities of spina bifida include hydrocephalus, motor function impairment, and bladder and bowel dysfunction [6].

Genetics and environmental factors have significant contributing roles in the etiology of NTDs [2,4,5,7,8]. Genetics is implicated as a causative factor in 20% of the cases, whereas the nongenetic elements account for 80% of NTD incidence [9]. Folic acid deficiency is a major environmental, and to a lesser extent genetic, contributor to the development of NTDs, specifically a folate (vitamin B9) deficiency in the mother from preconception through the first trimester [9,10]. The term “folate” is typically understood to refer to both folic acid in its synthetic form and natural folate [6].

Research performed in 1981 studied the effect of a daily dose of 4,000 μg of folic acid on NTDs [11]. Although it was a randomized study and the results reflected that protection by folic acid was provided against NTDs, the sample size was very small. In 1983, another study revealed that women with previous histories of pregnancies that resulted in children born with NTDs had a decreased risk of this occurring again if they consumed a multivitamin before and during the early part of later pregnancy. This group was compared to a similar group of women with the same history but who did not receive the multivitamin. However, this study was not randomized, and there was evidence of sample bias [12].

To determine the true effect of vitamins on the prevention of NTDs, the Medical Research Council (MRC) of the United Kingdom agreed to fund a randomized study with a large representative sample. This trial became known as the MRC Vitamin Study. Published in 1991, the results indicated that a 4,000-μg dose of a folic acid supplement significantly reduced the occurrence of births with NTDs [13]. To provide the benefits of folic acid to all childbearing women, many countries began fortifying foods with folic acid. In the United States, a program focused on fortifying cereal grain foods has been implemented since 1998.

The prevention of NTDs by administration of folic acid has been studied and discussed in the literature [2,4,5,7,8,14]. On an international level, at least 400 μg of folic acid has been recommended in the first month before conception and throughout the first trimester [15,16]. However, others have suggested 400 μg of folic acid as a daily dose for five to six months before pregnancy as it will take approximately 20 weeks to maintain the level needed to decrease the risk of NTDs [14]. Although many countries have been fortifying foods such as cereal grain products with folic acid, not all countries have adopted this practice [6]. As a key factor in the prevention of NTDs, this review will focus on the pharmacology of folic acid, neural tube formation, and the defects that can occur, specifically spina bifida, and folic acid’s relevance to the development of spina bifida. Current folate dosing as a vitamin supplement and integrated food fortification programs will also be discussed, and women’s knowledge and awareness of folic acid, along with its importance in the prevention of spina bifida, will be examined.

## Review

Pharmacokinetics and pharmacodynamics of folic acid and folate

Absorption and Metabolism of Folic Acid

**Figure 1:**
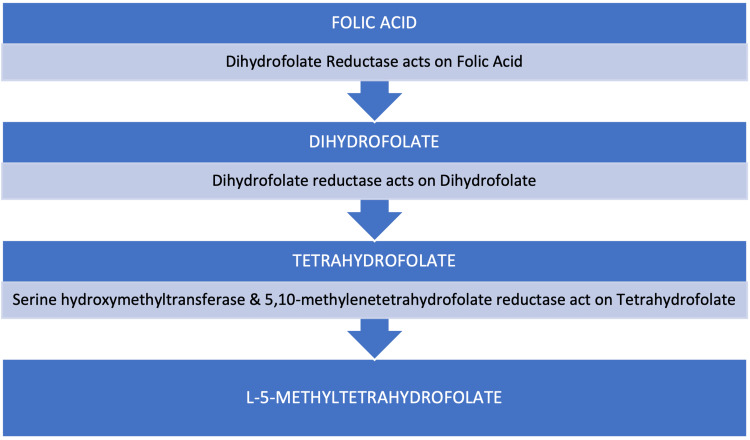
Metabolism of Folic Acid to L-5-Methyltetrahydrofolate After Intestinal Absorption.

Folate is the natural form of vitamin B9 that exists in foods such as broccoli, brussels sprouts, and other green leafy vegetables whereas folic acid is the synthetic form of folate that is often added to vitamins and foods such as cereal and grains. Folic acid exists as monoglutamate and can be readily absorbed by intestinal mucosal cells, whereas the dietary forms of folate, such as polyglutamate derivatives, are initially hydrolyzed by the brush border of the small intestine to achieve absorption. Since folates exist mostly in polyglutamate forms, they must be hydrolyzed into monoglutamates to be absorbed. This is achieved by intestinal glutamate carboxypeptidase II, also known as folate hydrolase. Once this has occurred, the monoglutamate forms can be transported across the mucosa [17]. Both folic acid and folate are absorbed in the proximal portion of the small intestine, specifically in the jejunum [18]. The proton-coupled folate transporter (PCFT) transports both oxidized as well as reduced folates with similar efficacy. Once the dietary forms are transported across the mucosa, they are metabolized into L-5-methyltetrahydrofolate. On the other hand, once inside the mucosal cell, folic acid is reduced to dihydrofolate (DHF) and then subsequentially tetrahydrofolate (THF) by the enzyme dihydrofolate reductase (DHFR). Once inside the mucosa cell, serine hydroxymethyltransferase and 5,10-methylenetetrahydrofolate reductase (MTHFR) metabolize folic acid to L-5-methyltetrahydrofolate [18,19], as seen in Figure 1.

Metabolism and Distribution of Folate

Once inside the bloodstream, L-5-methyl-THF exists in two forms: its free form and its form that loosely attaches to plasma proteins. It is carried to peripheral tissue via reduced folate carrier-1. This protein has been shown to have a poor affinity for folic acid, and it is more efficient for transporting reduced folates [20]. Folate is also transported via a second transporter called a folate-binding protein. This transporter has variable expression in tissues with high concentrations found in the choroid plexus, the placenta, the proximal tubules of the kidney, and some tumors [19]. The enzyme folylpolyglutamate synthetase plays a role in metabolizing folate from its folate monoglutamate form into polyglutamate. However, before this can occur, L-5-methylglutamate must be converted into THF via a reaction occurring because of methionine synthase. This reaction occurs with the help of cofactors B12 (cobalamin) as well as homocysteine. Once this reaction has occurred, the monoglutamate form can be converted into polyglutamate, allowing retention within the tissues [21]. There are mechanisms in place to prevent excessive amounts of folate from accumulating in tissues, mainly occurring when tissue folate levels reach an upper threshold. The competition for methionine synthase by L-5-methyl-THF polyglutamates and monoglutamates reduces the amount of L-5-methyl-THF [22]. For folic acid to become the metabolically active THF, it must undergo a two-step conversion conducted via DHFR. The first step is the conversion to DHF, which is the rate-limiting step. The second step involves the conversion of DHF to THF. THF can be converted as a one-carbon donor to different forms, including L-5-methyl-THF, 5,10-methylene-THF, or 5- or 10-formyl-THF, with L-5-methyl-THF being the most predominate form entering human metabolism. The THF forms can then be used as one-carbon donors in a variety of reactions [23].

Physiologic Actions of Folate

Folate is involved in many reactions that result in the production of DNA and RNA. These reactions include the methionine, the purine, and the thymidylate cycles. Folates provide a methylene moiety for the de novo synthesis of thymidylate from deoxyuridylate and two formate moieties for the de novo synthesis of the purine ring [24]. Thymidylate is required specifically for the synthesis of DNA. The enzyme thymidylate synthetase catalyzes the transfer of the one-carbon group from 5,10-methylene-THF to form deoxythymidine monophosphate (dUMP). This reaction oxidizes THF to DHF, and DHF must subsequently be reduced back to THF by the enzyme DHFR to be capable of being a carbon donor again [25]. A 5,10-methylene-THF can be used in the thymidylate cycle, reduced to L-5-methyl-THF to participate in methionine synthesis, or oxidized to 10-formyl-THF to participate in purine synthesis. Mammalian cells contain a large pool of folate in the mitochondria that can serve as a supply for reactions requiring one-carbon donor molecules [23]. Homocysteine is methylated to produce methionine and, in the process, uses L-5-methyl-THF as a carbon donor. Methionine can then be used in the form of S-adenosylmethionine as a methyl donor in the methylation of DNA, histones, neurotransmitters, phospholipids, and synthesis of creatine. These reactions are necessary for genomic stability and optimal gene expression [26].

Excretion of Folate

Folate is a vitamin that possesses long turnover and elimination rates, with a half-life that can be greater than 100 days in total [27]. Folate itself is minimally excreted in the urine and is only excreted in the feces to a small degree via bile. The vast majority of folate (over 99%) in the tissue exists in polyglutamate form. The catabolism of the polyglutamate form involves the cleavage at the C9-N10 bond, which generates p-aminobenzyl polyglutamate and a pterin moiety. P-aminobenzoyl polyglutamates are excreted in the urine as N-acetyl-aminobenzoylmoly glutamate, which is a product of a hydrolysis reaction catalyzed via the enzyme lysosomal glutamylhydrolase. Additionally, it is the pterin moiety that is excreted in the feces via bile [28].

Bioavailability of Folic Acid

Folate levels can be determined via serology by measuring the plasma levels, red blood cell (RBC) folate concentrations, and plasma homocysteine levels. Plasma folate levels are altered once folate is ingested [29]. Folate accumulates inside the RBCs during erythropoiesis. This serves as a marker of folate bioavailability over a longer period as RBC folate levels change slowly. Homocysteine and folate share an inverse relationship. When folate levels rise, homocysteine levels drop, and vice versa. This is because when folate and L-5-methyl-THF levels are low, there are minimal carbon donors available to aid in the conversion of homocysteine to methionine [30].

Spina bifida and folic acid deficiency

Neural Tube Formation and Defects

Defects of neural tube formation are the result of the abnormal development of the central nervous system within the embryo. During the third week of embryonic development, the process of gastrulation gives rise to the three germ layers within an embryo. One of these germ layers, the ectoderm, develops to form the neural plate. This neural plate undergoes further development through the process of primary and secondary neurulation, allowing for the development of the neural tube from the neural plate [8]. During primary neurulation, the neural plate develops into the cranial portion of the neural tube including the brain and spinal cord. During secondary neurulation, mesenchymal stem cell differentiation contributes to the development of the caudal portion of the neural tube including the sacral and coccygeal regions, which develop around the 26th day of gestation [31,32]. This process of neural tube development is regulated by signaling pathways within the notochord and surrounding tissue. These signaling pathways initiate the closure of the neural tube with multiple regions coming together simultaneously in a zipper-like process [8,33]. NTDs, a failure within the process of neural tube development, are further subdivided into two primary classes: closed and open defects; the latter of which is signified by the exposure of neural tissue and leakage of cerebrospinal fluid [8,31,34].

Spina bifida, defined by an abnormal opening (bifid) of the vertebral column, can be further subdivided into four defect-defined subclasses: occulta, closed spinal dysraphism, meningocele, and myelomeningocele [31]. The first class of spina bifida, occulta, is an asymptomatic, closed NTD that occurs because of a vertebral malformation allowing for an opening of the vertebral column. The defect is commonly covered by a layer of skin and sometimes accompanied by a hairy patch or dimple. The second class of spina bifida, closed spinal dysraphism, is another closed NTD consisting of a deficiency of at least two vertebral arches with malformations of fat, bone, or the meninges covering the spinal cord. The third class of spina bifida, meningocele, is a closed NTD that occurs because of the extrusion of a meningeal-enclosed pocket of cerebrospinal fluid through the posterior vertebrae or skull. A meningocele lacks all neural elements within the extrusion and may be exposed or covered by a layer of skin. The fourth class of spina bifida, myelomeningocele, may be an open or closed NTD that arises because of extrusion of the meninges, neural elements, and cerebrospinal fluid through the posterior vertebrae which may or may not be enclosed by the meninges [31,32]. Myelomeningocele is the most common and most severe type of spina bifida as the protruding neural tissues are exposed to amniotic fluid. This exposure can ultimately result in profound deficits such as motor, orthopedic, sensory, and urological dysfunction, fecal incontinence, and hydrocephalus [31,32].

Folic Acid Deficiency: Contributions to Neural Tube Defects

The primary medical approach to NTDs, including spina bifida, is prevention via a recommended dietary supplementation of folate (e.g., folic acid, vitamin B9) for women of childbearing age. Folic acid supplementation has been described to reduce the incidence rate of NTDs because of its major contributions to cell proliferation and neurulation [8]. Folate contributes to the synthesis of the nucleic acid building blocks, thymidylate, and purines, which can be found within the structure of DNA and RNA [23]. Folate also has been suggested to influence DNA methylation and histone modifications, thus playing a role in gene expression during neurodevelopment. Therefore, the absence of folate and its metabolites halts the proliferation and migration of neural tissue during neurulation, resulting in NTDs [8]. To address the link between low dietary folate levels and NTDs, many countries such as the United States, Canada, and Chile have begun establishing fortified foods with folic acid [35-37]. In countries where folic acid fortification was introduced and monitored, the rate of births with NTDs was subsequently reduced [6,8,36-38]. While the majority of NTD cases can be attributed to a lack of dietary folate in the absence of proper supplementation, some pregnant women may still give birth to children with NTDs because of genetic changes in the metabolism of folate, even with adequate supplementation before and during pregnancy. Defects in folate metabolism include the MTHFR C677T gene mutation, which decreases the activity of enzymes required for folate metabolism, thereby decreasing serum folate levels [1,39]. Exogenous folate antagonists inhibit folate absorption or increase the breakdown of folate, including phenytoin, carbamazepine, and other antiepileptic drugs. Other antagonists such as methotrexate, sulfasalazine, and trimethoprim decrease the activity of DHFR, preventing folate from converting into active metabolites [40]. Ultimately, this causes abnormal apoptosis of neural tissue contributing to NTD development [41].

Folic acid effects on the development of spina bifida

Folic Acid’s Mechanism of Action for Spina Bifida Prevention

The mechanism of action of folic acid on NTDs is not completely understood, but there are theories related to how folic acid can be protective during pregnancy. Studies have shown that pregnant women require folate at levels of 5-10 times greater than age-matched peers [42]. During pregnancy, there is a rapid formation and remodeling of organic materials. The development of these organic materials requires the creation of purines and pyrimidines, which are the nucleotide bases that make up DNA. Folic acid is transformed into 5,10-methylene-THF, an important source of carbon for the formation of nucleotide bases [43]. Higher levels of folic acid are required because of the need for carbon in the rapidly dividing cells of the embryo and subsequent developing fetus. At the most general level, it is believed that folic acid is protective against spina bifida and other NTDs as having adequate levels of folate ensures adequate availability of carbon [42]. The reason folate deficiencies present as NTDs specifically is because an enzyme complex called glycine cleavage system is present in large concentrations in the neuroepithelium [44]. Whenever folate is deficient, the glycine cleavage system is unable to create 5,10-methylene-THF, and, as a result, the creation of neural tissue stalls [44].

Dosage, Timing, and Methods of Folic Acid Administration to Prevent Spina Bifida

Starting in 1965, the medical community began investigating the link between folic acid and NTDs [45]. After promising results from trials in the following 25 years, government health agencies in the United States began advising reproductive-aged women to supplement their diet with folic acid [46]. The Centers for Disease Control and Prevention currently suggests a daily intake of 400 μg of folic acid [47]. To bring individuals closer to this minimum level, the United States began enriching grain with folic acid in 1998 [48]. Since the implementation of these requirements, 140 μg of folic acid have been added to every 100 grams of enriched grain [49]. Fortification serves as an important way to bolster intake, but women must consciously supplement their intake through a more direct method, especially in countries without stringent fortification protocols. Ideally, women would have daily intakes well above the 400-μg minimum [50]. When a woman is pregnant, the recommended level of daily folate intake rises to 600 μg, so many of the available oral prenatal vitamins include 0.8-1 milligram of folic acid to ensure adequate levels [51]. Unfortunately, it is common for women to initiate prenatal vitamin use only after discovering their pregnancy, often weeks after conception, by which time the crucial process of neurulation has already concluded. Neurulation is only a 28-day process; therefore, if a woman does not begin taking prenatal vitamins until she is one month pregnant, the critical period during which folic acid can be effective as a preventative for spina bifida will have been missed. Thus, it is recommended that all women of reproductive age maintain adequate folate levels through a balanced diet high in leafy green vegetables and enriched grains in combination with an oral folic acid supplement.

Women’s awareness/knowledge of folic acid in preventing spina bifida

Examination of Women’s Awareness/Knowledge of Folic Acid Supplements

As evidence continues to support the supplementation of folic acid during pregnancy, the general population must be educated on its effectiveness in preventing not only spina bifida but also other NTDs. There have been a multitude of studies on what the average reproductive-aged female knows about the benefits of folic acid supplementation, but their results have shown significant variation among demographic groups. Even among physicians, the knowledge of folic acid could be improved. The United States Preventative Services Task Force surveyed family medicine residents from 39 residency programs in 2020 and 2021 and questioned them about their knowledge of folic acid. This study found that 6.6% of the female resident physicians reported they “know a little” or “do not know” about the recommendations for folic acid intake [52]. In a study of 3277 women in Georgia who were pregnant during the years 2009-2011, it was found that over 73% of women knew the preventative capabilities of folic acid [53]. Nearly three-fourths of a population being educated on the necessity of folic acid supplementation is promising, but it is also important to investigate specific populations to identify groups at the highest risk of suboptimal education levels about this topic. A 2013 meta-analysis of studies involving women in the United Kingdom found that while over 80% of Caucasian women in one study were knowledgeable about folic acid’s benefits, less than 40% of Bangladeshi women were aware [54]. Another cited study found that 16% of pregnant women in their first trimester living in East London did not have any knowledge about folic acid or its benefits [54,55]. These results exhibit the necessity for increased educational initiatives, especially in areas of high incidence of NTDs. In a more promising study released in 2021, over 80% of pregnant women polled in China understood that folic acid was protective against the development of NTDs [56]. In another study, there was a positive relationship between counseling about folic acid before conception and increased use of folic acid. However, it was noted that most of the women in the study did not receive counseling [57]. An examination of women of childbearing age in the United States revealed a reduction in the oral intake of a multivitamin containing folic acid from the years 2006-2016, reflecting the importance of adequate messaging in educational programs to increase awareness [58]. Many of the respondents in various studies have attributed their knowledge to the media and their healthcare professionals. Therefore, it is important to consider ways in which these avenues can be better utilized to advocate for the need for adequate folic acid intake in all women of childbearing age. In Table 1, additional studies related to knowledge of folic acid in Saudi Arabia, Nigeria, Poland, Ukraine, and Ethiopia have been summarized.

**Table 1:** Knowledge of Folic Acid Among Reproductive-Aged Women in Five Different Countries. FA: folic acid

Author (Year)	Patient Population/ Purpose of Study	Results/Findings	Conclusions
Sabi et al. (2022) [59]	Female undergraduate students in Saudi Arabia/Measure knowledge of FA and supplements	61% (266/437) who had heard of or had knowledge of FA 55.3% (241/437) were aware of the timing of FA intake. 55.7% (243/437) knew of the FA duration of intake 83% (362/437) knew diseases prevented by FA.	A large percentage of college-age women are not knowledgeable about FA and its intake requirements. Educational outreach in schools, physician’s offices, and communities are needed (e.g., health clinics).
Okon et al. (2020) [60]	Women of childbearing age in Nigeria receiving services in healthcare facilities/Determine awareness and use of FA	48% (281/586) were aware of FA as a supplement. 30.4% (178/586) knew of nutritional sources of FA. 25% (152/598) knew of FA benefits. 37.7% (221/586) used FA. 2.4% (14/586) used FA in 1st trimester.	Low level of FA knowledge as a supplement, its sources/benefits; very low level of use in the first trimester; Educational programs needed in schools, physician’s offices, and communities (e.g., health clinics) to increase the knowledge and use of FA.
Zadarko-Domaradzka et al. (2021) [61]	Women of childbearing age in Poland/Assess awareness of FA recommendations and FA effectiveness	14% (178/1285) used FA supplements. 52.4% (673/1285) planned to take FA for pregnancies. 55.5% (713/1285) said daily FA was unnecessary.	Extremely low level of FA use. Educational outreach is needed in schools, physician’s offices, and communities (e.g., health clinics).
Hlushko et al. (2021) [62]	Medical students (76.3% female) in Ukraine/Examine awareness, knowledge, effects of FA, and knowledge of NTDs	86.8% (99/114) knew FA deficiency caused congenital defects. 67.5% (77/114) knew FA was essential during pregnancy. 53.5% (61/114) knew to take it preconception.	Low level of knowledge among medical students regarding FA importance during preconception and pregnancy. The Curriculum needs to address FA’s significance to prevent NTD and its necessity before and during pregnancy.
Begashaw et al. (2022) [63]	Ethiopian women of childbearing age/assess the knowledge for the need of FA during preconception	35.1% (155/441) knew preconceptional FA could prevent NTDs. 3.8% (17/441) knew when to take preconceptional FA.	Low level of knowledge regarding the need/timing of taking FA supplementation. Targeted educational outreach programs regarding the benefits of daily FA intake, especially during preconception and pregnancy, are needed.

## Conclusions

As the most common central nervous system birth anomaly, spina bifida continues to occur worldwide. This NTD presents lifelong challenges that can have a profound effect on a child’s quality of life. The dynamics of the child’s family life will also be affected depending on the severity of the illness and developmental disabilities that ensue. As reflected in this review, various international studies have demonstrated a reduction in the incidence of spina bifida with the appropriate dose and timing of folic acid administration. All women must be provided equal access to folic acid via vitamins or a food supplementation program to decrease the risk of NTDs. Moreover, it is important to emphasize the need for all women of reproductive age to make a conscious effort to include sufficient folic acid in their daily diet whether that be through vitamins and/or food supplementation.

The other essential element to outreach is the education of women and their communities regarding the link between folic acid deficiency and spina bifida. Implementation can occur in healthcare facilities, physician offices, schools, and on the Internet. The inclusion/accessibility of folic acid as a vitamin or food supplement should be coupled with educational programs that address the importance of folic acid and its significance as a deterrent to the development of spina bifida. Further research should be conducted to determine the effectiveness of such programs when evaluating folic acid use among women, especially those of reproductive age. It is noteworthy that there is a paucity of research regarding folic acid awareness, knowledge, and intake in the United States in the latter half of the 2000s to the present time. As a country where folic acid is available as a vitamin and in food fortification programs, data regarding factors that influence the use of folic acid are important to the reduction in births with NTDs. Although some women will deliver newborns with this congenital anomaly despite receiving and maintaining an appropriate level of folic acid, there remains a decreased risk of having a child with spina bifida and other NTDs.
